# N^6^ -Methyladenosine Negatively Regulates Human Respiratory Syncytial Virus Replication

**DOI:** 10.3389/fcell.2021.739445

**Published:** 2021-10-04

**Authors:** Fabian Figueroa, Alonso Vega-Gibson, Joseline Catrileo, Aracelly Gaete-Argel, Sebastian Riquelme-Barrios, Luis Antonio Alonso-Palomares, Lorena I. Tapia, Fernando Valiente-Echeverría, Ricardo Soto-Rifo, Monica L. Acevedo

**Affiliations:** ^1^Laboratory of Molecular and Cellular Virology, Virology Program, Institute of Biomedical Sciences, Faculty of Medicine, Universidad de Chile, Santiago, Chile; ^2^HIV/AIDS Workgroup, Faculty of Medicine, Universidad de Chile, Santiago, Chile; ^3^Department of Pediatrics and Pediatric Surgery, Hospital Roberto del Río, Faculty of Medicine, Universidad de Chile, Santiago, Chile

**Keywords:** human respiratory syncytial virus, N^6^-methyladenosine, RNA modification, inclusion bodies, gRNA synthesis, viral replication

## Abstract

N^6^-methyladenosine (m^6^A) is the most abundant internal modification described in eukaryotic mRNA and several viral RNA including human respiratory syncytial virus (HRSV). Here, we evaluated the impact of m^6^A writers, erasers and readers on HRSV genomic RNA accumulation and inclusion bodies assembly during viral replication. We observed that the METTL3/METTL14 m^6^A writer complex plays a negative role in HRSV protein synthesis and viral titers, while m^6^A erasers FTO and ALKBH5 had the opposite effect. We also observed that m^6^A readers YTHDF1-3 bind to the viral genomic RNA inducing a decrease in its intracellular levels and thus, inhibiting viral replication. Finally, we observed that overexpression of YTHDFs proteins caused a decrease in the size of inclusion bodies (IBs), accompanied by an increase in their number. METTL3 knockdown cells showed an opposite effect indicating that the dynamics of IBs assembly and coalescence are strongly affected by m^6^A readers in a mechanism dependent on m^6^A writers. Taken together, our results demonstrated that the m^6^A modification negatively affects HRSV replication, possibly through a mechanism involving the assembly of inclusion bodies, the main factories of viral genomic RNA synthesis.

## Introduction

Human respiratory syncytial virus (HRSV) infections are the main cause of viral bronchiolitis and pneumonia in infants, elderly, asthmatic and immunocompromised individuals worldwide reaching 33 million cases annually. HRSV is an enveloped virus belonging to the genus *Orthopneumovirus* of the *Pneumoviridae* family and possesses a 15-kb negative-stranded RNA as a genome (Afonso et al., 2016; Rima et al., 2017). The viral replication occurs in inclusion bodies (IBs), defined as membrane-less ribonucleoprotein complexes containing the genomic RNA (gRNA) together with viral proteins: nucleoprotein (N), phosphoprotein (P), matrix (M), nucleocapsid-associated transcription factor (M2-1) and the large polymerase subunit (L) (Norrby et al., 1970; García-Barreno et al., 1996; Ghildyal et al., 2005; Carromeu et al., 2007; Li et al., 2008; Lindquist et al., 2010). As such, IBs are the sites where viral mRNA and gRNA synthesis occurs, but they also contribute to avoiding the recognition of viral components by the innate immune system (Lifland et al., 2012; Rincheval et al., 2017).

N^6^-methyladenosine (m^6^A) is the most abundant internal modification described in eukaryotic mRNA and viral RNA and has been involved in regulating multiple cellular and pathological processes, including viral infections (Lichinchi et al., 2016a; Courtney et al., 2017; Imam et al., 2018; Lu et al., 2018; Hao et al., 2019; Liu et al., 2019; Price et al., 2020; Wu et al., 2020). The cellular machinery responsible of this regulation includes the complex formed by methyltransferases METTL3 and METTL14, which introduce the methyl group at the N^6^ position of adenosines mainly located in the DRACH context (Liu et al., 2014), FTO (Jia et al., 2011), and ALKBH5 (Zheng et al., 2013), which have been shown to reverse adenosine methylation and YTH proteins that read the modification and are responsible for exerting the functions of m^6^A (Meyer and Jaffrey, 2014).

Several groups have observed that the m^6^A modification has a relevant role during the replication cycle of viruses that replicate both in the nucleus and the cytoplasm (Williams et al., 2019; Wu et al., 2019). For instance, during hepatitis C virus (HCV) infection, YTHDF proteins relocate to lipid droplets, sites where viral RNA synthesis and viral particle assembly take place, to interfere with viral RNA packaging (Gokhale et al., 2016). During Zika virus (ZIKV) replication, a decrease in viral RNA levels induced by YTHDF proteins was described (Lichinchi et al., 2016b). Similarly, YTHDF proteins decrease the levels of the incoming genomic RNA at the early stages of HIV-1 replication (Lu et al., 2018) but at post-integration steps, the binding of YTHDF1-3 to the 3′-UTR of the viral RNA results in the accumulation of viral transcripts with the concomitant increase in protein synthesis (Kennedy et al., 2016; Tirumuru et al., 2016). The presence of m^6^A has also been involved in the recognition of the viral RNA by the immune system. As such, several reports have shown that m^6^A allows viral RNA to escape recognition by sensors such as RIG-I and MDA5 (Kim et al., 2020; Lu et al., 2020; Qiu et al., 2021).

The m^6^A modification has also been found in HRSV and the closely related human metapneumovirus (HMPV), in both viruses, the m^6^A modification was identified in viral mRNA as well as in genomic and antigenomic RNA (Xue et al., 2019; Lu et al., 2020). In HeLa cells, the stable overexpression of YTHDF1, 2 and 3 was shown to favor the expression of viral proteins and genomic RNA levels and viral titers from the first 12 h of replication. Further, overexpression of the METTL3/METTL14 proteins also increased m^6^A levels of the viral RNA increasing the expression of the F and G viral proteins. In contrast, overexpression of the FTO and ALKBH5 was shown to exert the opposite effect (Xue et al., 2019; Lu et al., 2020).

In this work, we analyzed the role of the cellular m^6^A machinery on genomic RNA abundance and replication of HRSV in HEK293T cells. Contrary to what was reported in HeLa cells, we observed that the METTL3/METTL14 complex plays a negative role in viral replication by inhibiting viral titers, viral protein synthesis and genomic RNA levels. However, we also observed that this regulation is dynamic and can be reversed by overexpression of ALKBH5. Strikingly, we observed that overexpression of YTHDFs decreases the size but increases the number of IBs in cells infected with HRSV in a process requiring METTL3. These data strongly suggest that m^6^A readers affect the dynamics of inclusion bodies assembly, possibly preventing their fusion and interfering with HRSV replication in HEK293T cells.

## Materials and Methods

### Cell Culture, Transfection and Infection

HEK293T (ATCC, CRL-11268) and MA-104 (ATCC, CRL2378.1) cells were maintained in Dulbecco’s modified Eagle’s medium (DMEM) supplemented with 10% inactivated fetal bovine serum (HyClone), 100 U/mL penicillin and 100 μg/mL streptomycin. The cells were grown in a humidified atmosphere with 5% CO_2_ at 37°C. The overexpression of the m^6^A machinery was performed by transient transfection of HEK293T cell with plasmid DNA using the FuGENE^®^ 6 System (Promega) according to manufacturer’s protocol with the following plasmids: pcDNA-Flag-METTL3 and pcDNA-Flag-METTL14, p3XFlag-CMV-FTO, pEGFP-CIB-ALKBH5, pcDNA-Flag-YTHDF1, 2, 3 or vectors expressing d2EGFP in the same backbone as control (Addgene). Six hours post-transfection the cells were infected with HRSV strain Tracy at a MOI:1 for 24 h.

### Generation of METTL3 Knockdown Cell Line

Lentiviral particles were produced in HEK293T cells. In brief, cells were co-transfected with VSV-G-0.8 μg (Addgene), PsPax2-1.5 μg (Addgene #12260) and METTL3-1.5 μg or pLOK.1-1.5 μg as a control, containing the shRNA sequence targeting (Sigma-Aldrich) using linear PEI protocol 25,000 Da (Polysciences) prepared as described previously (Toro-Ascuy et al., 2018). Cells were maintained in DMEM (Life Technologies) supplemented with 10% FBS (Sigma-Aldrich) at 37°C with 5% CO_2_ atmosphere. Supernatants were collected 48 hours post-transfection, cleared through a 0.22 μm filter, and employed to transduce 5 × 10^5^ HEK293T cells/well. The medium (containing lentiviral particles) was replaced by fresh DMEM containing puromycin (1 μg/mL; Sigma-Aldrich) at 24 hours post-transduction, the cells were selected for 10 days at 37°C and 5% CO_2_. METTL3 expression was evaluated by Western blot assay.

### Human Respiratory Syncytial Virus Titration Assay

Infective titer of HRSV was measured by 10-fold serial dilutions in DMEM containing 2% fetal bovine serum (DMEM-2%). 100 μl of each dilution was absorbed in triplicate for 90 min on 96-well plates of MA-104 cells and incubated with DMEM-2% for 8 days. The supernatant was recovered, the cells were fixed with 3.7% formaldehyde and stained with 0.1% crystal violet. Only wells where the cytopathic effect appears in more than 50% of inoculated tissue culture cells were considered. TCID_50_/mL was calculated following Spearman-Karber formula (Ramakrishnan, 2016): M = xk + d [0.5-(1/n) (r)], where xk: a dose of the highest dilution; R: sum of the number of negative responses; d: spacing between dilutions; n: wells per dilution.

### RNA Extraction and RT-qPCR

Total RNA was extracted using TRIzol^®^ (ThemoFisher) following the manufacturer’s instructions. Subsequently, a negative strand-specific amplification was performed by reverse transcription coupled to real-time PCR as described in Bannister et al. (2010) with some modifications. First, 1 μg of cytoplasmic RNA was reverse transcribed with the High Capacity RNA-to-cDNA^TM^ kit (ThermoFisher) using 25 U of MultiScribe Reverse transcriptase, 0.5 mM dNTP mix, and 2.5 μM specific primer RNA-F(-)HRSV/RT: CGGTCATGGTGGCGAATAAATTGATCAATGATATGCCTAT AA containing a tag and primer reverse of HMBS or GAPDH. The reaction was performed at 25°C for 10 min followed by 40 min at 48°C and 5 min at 95°C. In the second reaction, the qPCR was performed using 250 nM of TATTATAGACATGATAGAATAACTTTG and the tag-specific primer CGGTCATGGTGGCGAATAA, 1X Brilliant SYBRGreen (Agilent Technologies) and 2 μL of 1:10 of cDNA. HMBS or GAPDH were used as normalization of cytoplasmic RNA extraction. qPCR was performed using the AriaMX Real Time PCR System (Agilent Technologies) with the following amplification parameters: initial denaturation at 95°C for 10 min and 50 cycles of denaturation at 95°C for 15 s, annealing at 50°C for 30 s and elongation at 72°C for 30 s. Fluorescence data were collected during the elongation step. Pfaffl formula (Pfaffl, 2001), which considers the amplification efficiency of each primer, was used for relative quantification.

### Western Blot Analysis

Cells were lysed in RIPA buffer supplemented with protease inhibitors cocktails (Roche) and 20 μg of total protein were subjected to 12% SDS polyacrylamide gel electrophoresis. The samples were transferred to Amersham Hybond^TM^ -P membrane (GE Healthcare) and blotted using anti-F (SantaCruz, 1:500); anti-Flag (BioLegend, 1:5,000); anti-GFP (SantaCruz, 1:5,000), anti-METTL3 (Cell Signaling Technology, 1:1,000) and anti-GAPDH (SantaCruz, 1:5,000). A corresponding horseradish peroxidase (HRP)-conjugated antibody (Jackson ImmunoResearch, 1:10,000) was used as a secondary antibody. The membranes were analyzed with the ECL substrate (Cyanagen) using a MiniHD9 scanner (Uvitec).

### Immunoprecipitation

HEK293T cells were transfected with pcDNA-Flag-YTHDF1, 2, 3 or pcDNA-d2EGFP as control and infected with HRSV as described above. At 24 hours post-infection, cells were washed in PBS and lysed in 1:1 of polysomes lysis buffer [100 mM KCl; 5 mM MgCl_2_; 10 mM Hepes pH 7.0; 0.5% Nonidet P-40; 1X Protease Inhibitor, 2 mM RNase Inhibitor (VRC) and 1 mM Dithiothreitol (DTT)] for 10 min on ice and centrifuged at 10,000 *g* for 15 min at 4°C. Cell extracts were incubated with previously washed Anti-Flag^®^ M2 Magnetic Beads (Sigma Aldrich) overnight at 4°C. The beads were washed five times with radioimmunoprecipitation buffer (Tris–HCl 50 mM pH 7,4; NaCl 150 mM, MgCl_2_ 1 mM; 0,05% Nonidet P-40), and the beads were separated in two. For Western blot, the samples were eluted with load buffer at 95°C for 5 min and for RT-qPCR, the RNA was extracted with Trizol^®^ following the manufacturer’s instructions. Then HRSV gRNA was amplified by RT-qPCR as detailed above.

### Immunofluorescence and Confocal Microscopy

Fifty thousand HEK293T cells were seeded on a coverslip in 12-well plates one day before the transfection was performed, then the cells were transfected with 1 μg of plasmid pcDNA-Flag-YTHDF1, 2 or 3, pcDNA-METTL3 or pcDNA-METTL3-D395A using linear polyethylenimine (PEI) as described in Fröhlich et al. (2016) and six hours later, the cells were infected with HRSV (MOI:1). Knockdown cells were prepared as before. At 24 hours post infection (hpi), cells were fixed with PBS-formaldehyde 4%, washed and permeabilized with Triton X-100 0.1%. After the blockade with BSA 1%, the cells were incubated with mouse anti-NP (sc-58001, Santa Cruz Biotechnologies, 1:250) and rat anti-Flag (637303, BioLegend, 1:250) for 1 h at 37°C. Then the cells were washed for 10 min in PBS followed by staining for 1 h at 37°C with anti-mouse Alexa Fluor 594 and anti-rat Alexa Fluor 488 (Molecular Probes) diluted 1:500. After three washes with PBS, the cells were incubated with DAPI (0.3 μg/mL in PBS) (Life Technologies) for 1 min at room temperature and the coverslips were mounted with Fluoromount^TM^ (Sigma-Aldrich). Cells were analyzed on an Olympus IX73 inverted microscope or a Carl Zeiss LSM700 confocal microscopy, where image acquisitions of multi-labeled cells were performed. For quantification, twenty independent Z-stack at zoom X2.5 of each experimental condition were taken. Image analysis was performed with the Image J software.

### Statistical Analysis

Data are presented as mean ± SD. The statistical analysis was performed using *t*-test in GraphPad Prism 6.0. *P*-value < 0.05 was considered statistically significant.

## Results

### METTL3/METTL14 Complex Inhibits Human Respiratory Syncytial Virus Replication in HEK293T Cells

**FIGURE 1:**
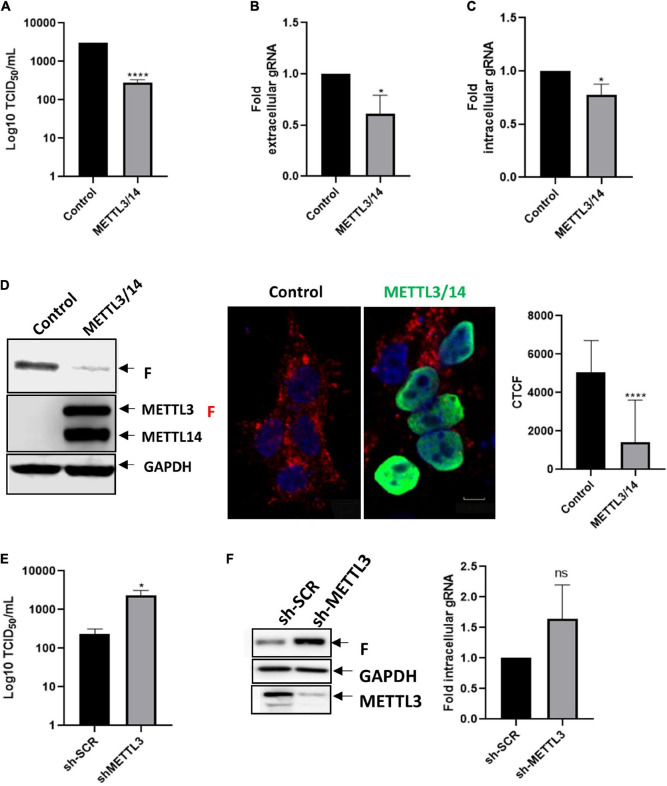
METTL3/METTL14 complex regulates the replication and gRNA synthesis **of** HRSV. **(A)** HEK293T cells were transfected with vectors expressing Flag-tagged METTL3 and METTL14 or a control vector expressing d2EGFP. Then the cells were infected with HRSV at MOI:1 for 24 h. The supernatant was recovered, and the infectious titer was analyzed by the TCID_50_ assay. **(B)** The extracellular and **(C)** intracellular gRNA was quantified by RT-qPCR. Results were normalized vs. the control (arbitrary set to 1) and expressed as the mean ± SD of three independent experiments. **(D)** Cell lysates from infected cells were used for the detection of the F protein and Flag-tagged proteins by Western blot. GAPDH was used as a loading control. The expression of F protein (red), METTL3/METTL14 complex (green) and nuclei (DAPI, blue) were analyzed by indirect immunofluorescence; representative fields are shown. Scale bar: 50 μm. Corrected total cell fluorescence (CTCF) of F protein (red) from thirty cells was quantified using Image J. **(E)** Control and METTL3 knock down HEK293T cells were infected with HRSV at MOI:1 for 24 h, the supernatants were collected, and infectious titer was analyzed by the TCID50 assay. **(F)** The F protein was analyzed by Western blot and intracellular levels of the gRNA were quantified from control and METTL3 knock down cells. Results are representative of three independent experiments (*p < 0.05; ****p < 0.0001; ns, not significant).

To evaluate the impact of the host m^6^A machinery on HRSV replication, we first analyzed the role of the methyltransferase complex on the replication of HRSV by overexpressing Flag-tagged METTL3 and METTL14 in HEK293T cells. We analyzed the viral titer through the TCID_50_ assay and observed an 11-fold decrease compared to the control suggesting a negative effect of m^6^A on viral production (Figure 1A). Although we observed a slight but significant decrease in the extracellular genomic RNA it does not explain the strong decrease in viral titers (Figure 1B). Consistent with this observation, we also observed a slight but significant decrease in the intracellular levels of the genomic RNA (Figure 1C) suggesting that m^6^A writers marginally affect genomic RNA synthesis and/or stability. However, analysis of the intracellular levels of the F protein revealed a dramatic decrease (Figure 1D) suggesting a major impact of METTL3/METTL14 overexpression on viral protein synthesis. To further confirm these observations, we knocked down endogenous METTL3 in HEK293T cells using shRNA. In agreement with the data presented above, the viral titer increased by 11-fold in METTL3 knockdown cells compared to the control (Figure 1E). Moreover, we observed an important increase in the levels of the F protein and a slight but non-significative increase in the genomic RNA levels (Figure 1F). Taken together, our results suggest that the m^6^A methyltransferase complex restricts HRSV replication by at least interfering with F protein synthesis and the production of infectious particles.

### FTO and ALKBH5 Enhance the Synthesis of the Human Respiratory Syncytial Virus F Protein

**FIGURE 2:**
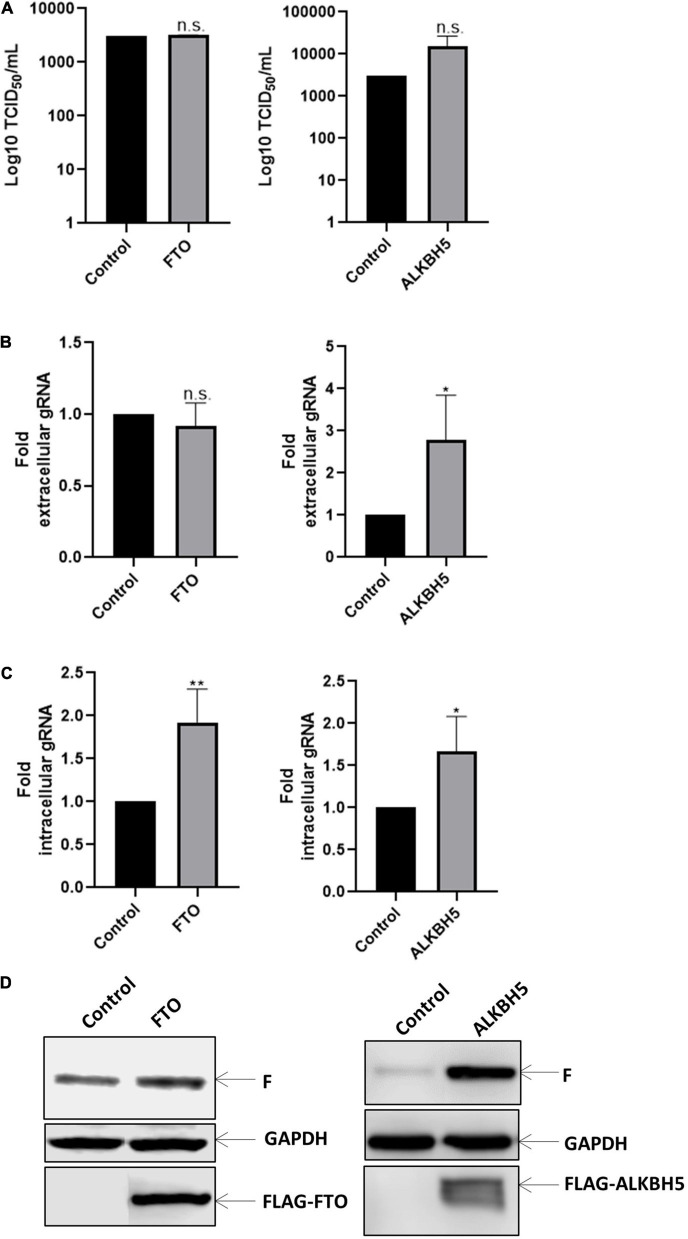
Erasers proteins favor gRNA synthesis. HEK293T cells were transfected with the vector expressing Flag-tagged FTO or ALKBH5 a control vector expressing d2EGFP. Then the cells were infected with HRSV at MOI:1 for 24 h. **(A)** The supernatant was recovered, and the infectious titer was analyzed by the TCID_50_ assay. **(B)** The extracellular and **(C)** intracellular gRNA was quantified by RT-qPCR. Results were normalized vs. the control (arbitrary set to 1) and expressed as the mean ± SD of three independent experiments. **(D)** Cell lysates from infected cells were used for the detection of the F protein and Flag-tagged proteins by Western blot. GAPDH was used as a loading control (*p < 0.05; **p < 0.01; ns, not significant).

Then, we sought to evaluate whether the negative effect of the m^6^A writer complex could be reversed by the m^6^A erasers FTO and ALKBH5. Although we observed an important effect on viral titers upon METTL3/METTL14 overexpression and METTL3 knockdown, our data indicate that overexpression of RNA demethylases FTO and ALKBH5 has not a significant impact on viral titers (Figure 2A). Nevertheless, we observed that extracellular genomic RNA levels increased under ALKBH5 but not FTO overexpression, suggesting a small increase in viral release that is not sufficient to significantly increase viral titers (Figure 2B). We also observed a 1.9 and 1.7-fold increase in the intracellular genomic RNA levels upon FTO and ALKBH5 overexpression, respectively (Figure 2C). Interestingly and in agreement with what was observed under METLL3 knockdown, we observed an increase in the expression of the F protein when both demethylases were overexpressed with a more prominent effect of ALKBH5 (Figure 2D). Together, these results suggest that FTO and ALKBH5 favor the synthesis of the HRSV F protein.

### YTHDF1-3 Proteins Decrease the Levels of the Human Respiratory Syncytial Virus Genomic RNA

**FIGURE 3:**
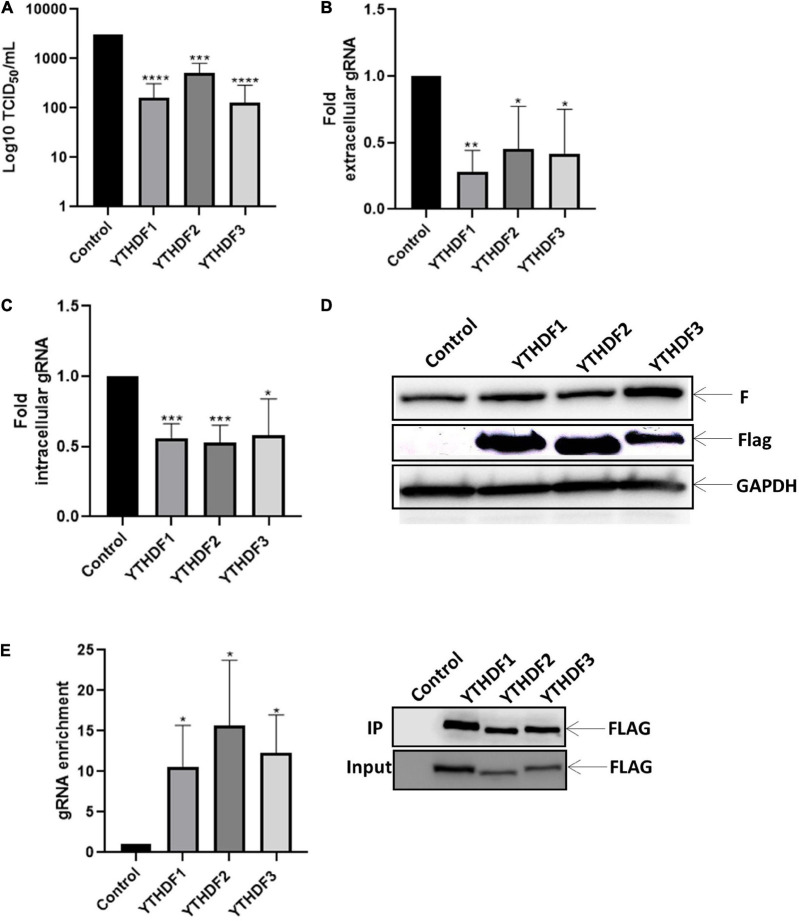
YTHDF proteins bind to the gRNA and disadvantage the synthesis of gRNA along with viral replication. HEK293T cells were transfected with the vector expressing YTHDF1-3 and, the control vector expressing d2EGFP. Then the cells were infected with HRSV at MOI:1 for 24 h. **(A)** The supernatant was extracted, and the infectious titer was analyzed by the TCID_50_ assay. **(B)** The extracellular and **(C)** intracellular gRNA was quantified by RT-qPCR. Results were normalized vs. control (arbitrary set to 1) and expressed as the mean ± SD of three independent experiment. **(D)** Cell lysates from infected cells were used for the detection of the F protein and Flag-tagged proteins by Western blot. GAPDH was used as a loading control. **(E)** Enrichment of gRNA by immunoprecipitation anti-Flag from cell extracts. gRNA was quantified by RT-qPCR and the fold of enrichment was graphed in relation to the control. The data are representative of three independent experiments (*p < 0.05; **p < 0.01; ***p < 0.001; ****p < 0.0001; ns, not significant).

Next, sought to evaluate whether the effects of m^6^A observed above were exerted by m^6^A readers. For this, we analyzed the impact of YTHDF1, 2 or 3 on viral replication. Interestingly, we observed that overexpression of YTHDF proteins indistinctly exerted a negative role on viral titers as well as in the extracellular and intracellular levels of the genomic RNA (Figures 3A–C). However, we observed that overexpression of m^6^A readers have no impact on the F protein levels (Figure 3D), indicating that the genomic RNA is the major target for these m^6^A readers. Consistent with a redundant negative role of YTHDF proteins on intracellular HRSV genomic RNA levels, RNA immunoprecipitation analyses indicate that the three m^6^A readers bind the HRSV gRNA indistinctly (Figure 3E). Together, these data indicate that YTHDF proteins indistinctly bind to the HRSV genomic RNA to interfere with viral production.

### YTHDF Proteins Decrease the Size of Inclusion Bodies

**FIGURE 4:**
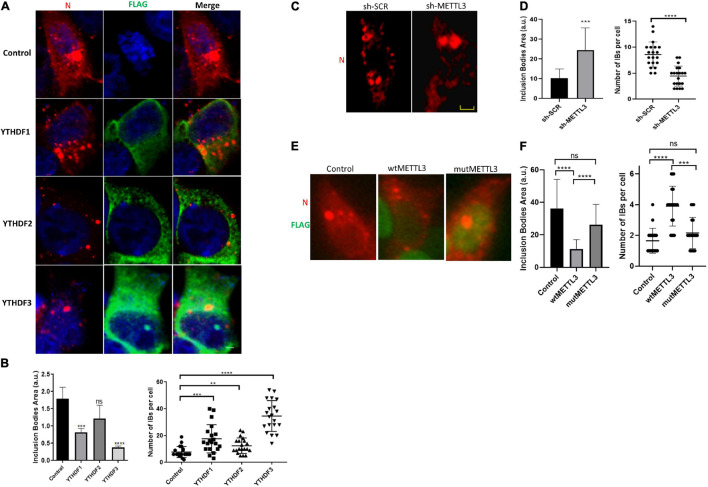
YTHDF proteins decrease in size but increase the number of inclusion bodies during HRSV infection. **(A)** Fluorescent photomicrographs of HEK293T cells transfected with vector expressing YTHDF proteins and infected with HRSV at MOI:1 for 24 h. Antibody anti-Flag (green) was used for immunostained YTHDF proteins and HRSV N (red). Nuclei (blue) was labeled with DAPI. **(B)** The area and number of IBs were quantified using Image J. **(C)** The localization of N protein (red) was analyzed by indirect immunofluorescence in HEK293T in which METTL3 was knocked down and shRNA control infected with HRSV at MOI:1 for 24 h. **(D)** The area and number of IBs were quantified using Image J. **(E)** The localization of N protein (red) and Flag (green) of HEK293T transfected with vector expressing wild-type or D395A METTL3 was analyzed by immunofluorescence. **(F)** The area and number of IBs were quantified using Image J. Data represent the mean ± SD (*n* = 20 cells per condition). Scale bar: 20 μm. The statistical significance was calculated, where: **p < 0.01; ***p < 0.001; ****p < 0.0001; ns, not significant.

We finally wanted to look at the mechanism by which YTHDF proteins were interfering with genomic RNA levels and viral titers through the analysis of inclusion bodies assembly under overexpression of YTHDF1-3. First, we observed that none of the m^6^A readers was recruited to IBs (Figure 4A). Interestingly, while overexpression of YTHDF1, YTHDF2 and YTHDF3 resulted in a decrease of the size of inclusion bodies at 24 hpi, only the effects of YTHDF1 and YTHDF3 were significant (Figure 4B). It is noteworthy that the reduction in IBs size was accompanied by an increase in their number suggesting that the dynamics and coalescence of inclusion bodies were affected by overexpression of the m^6^A readers (Figure 4B).

Finally, to assess whether these changes in IBs coalescence were due to the methylation of the viral RNA by the m^6^A writer complex, we analyzed IBs number and size in control and METTL3 knockdown cells. Interestingly, we observed a significant increase in the size and a decrease in the number of IBs under METTL3 knockdown (Figures 4C,D). In contrast, we observed a significant decrease in the size and an increase in the number of IBs upon METTL3 overexpression. Such an effect was not observed upon overexpression of a catalytically inactive mutant (METTL3-D395A) indicating that IBs dynamics and coalescence depends on the catalytic activity of the m^6^A writer complex (Figures 4E,F, compare wtMETTL3 and mutMETTL3).

Together, these data suggest that methylation of the HRSV genomic RNA by METTL3 and its binding by the YTHDF proteins leads to an inhibition of the coalescence of inclusion bodies resulting in decreased viral titers.

## Discussion

Different studies have shown that the m^6^A modification impacts viral replication through the regulation of viral RNA metabolism. However, these effects are diverse depending on the virus and the cell type used and thus, the molecular mechanisms underlying the epitranscriptomic regulation of viral replication need more exploration. Our study determined the effects of the m^6^A machinery at different stages of HRSV replication in HEK293T cells. We observed that m^6^A writers negatively impact the synthesis of the viral protein F in a process that can be reversed mainly by the RNA demethylase ALKBH5. On the other hand, we observed that YTHDF proteins bind to the HRSV genomic RNA inducing a decrease in the intracellular levels of the viral genome. We also observed that YTHDF proteins overexpression resulted in a decrease in the size of IBs together with an increase in their number. The opposite effect was observed when we knocked down METTL3 suggesting a role for the methylation of the viral RNA and its recognition by YTHDF proteins in the coalescence of HRSV IBs.

Several regulatory functions on cellular and viral RNA metabolism have been attributed to the m^6^A modification in the cytoplasm ranging from translation to localization and stability. Interestingly, Xue et al. (2019) detected m^6^A peaks in the HRSV negative-sense genomic RNA and the positive-sense antigenomic RNA obtained from purified viral particles from the supernatants of infected HeLa cells, particularly in the regions of the N, P, G, and F genes. In addition, the authors identified that HRSV mRNAs including N, P and G but not F also contained m^6^A sites although the m^6^A machinery positively regulated F protein levels in HeLa cells. In this way, our data suggest that the presence or absence of m^6^A within the HRSV F mRNA in HEK293T cells affects viral fusion protein levels with consequences in the release of infectious viral particles. An antiviral effect due to m^6^A readers has also been observed in positive-strand RNA viruses such as ZIKV and HCV (Gokhale et al., 2016). We observed that the negative impact of m^6^A on F protein expression was reversed by METTL3 knockdown and ALKBH5 overexpression. Since no effects on F protein levels were observed upon overexpression of YTHDF1, 2 or 3, it is tempting to speculate that other cytoplasmic m^6^A reader such as YTHDC2 could be involved in this regulation. Indeed, YTHDC2 has been involved in the regulation of translation and stability of m^6^A-modified mRNAs (Kretschmer et al., 2018).

Interestingly, METTL3 knockdown but not ALKBH5 overexpression resulted in a significant increase in the production of infectious viral particles. These observations suggest that methylation of other viral RNAs (such as mRNAs, the genomic and/or antigenomic RNA) that are not substrate for ALKBH5 are involved in producing infectious virions. Consistent with this notion, we observed that YTHDF1, 2 and 3 bind the HRSV genomic RNA, inducing a decrease in the intracellular and extracellular levels of the viral genome with the consequent reduction of viral titers. Whether other m^6^A methyltransferases acting on other RNA molecules such as METTL16 (U6 snRNA), METTL5 (18S rRNA), ZCCHC4 (28S rRNA) or METTL4 (U2 snRNA) (Pendleton et al., 2017; Ren et al., 2019; van Tran et al., 2019; Gu et al., 2020) play a role in HRSV replication requires further investigation.

The negative impact of the m^6^A modification and YTHDF proteins on IBs assembly has not been described before and appears to have an important effect on the dynamics of assembly or coalescence of these cytoplasmic structures, where small IBs can merge and become larger (Cifuentes-Muñoz et al., 2017). Although the mechanisms of HRSV IBs formation are poorly understood, it has been established that they correspond to highly dynamic structures undergoing a dramatic reorganization during the viral replication cycle. IBs are active viral replication sites where interactions between the viral proteins N and P are the minimal requirement for their formation. In this sense, YTHDF proteins seem to be exerting their role by altering the coalescence of IBs, decreasing the synthesis of the genomic RNA and, consequently viral replication. Since we could not observe the colocalization of YTHDF proteins with IBs, the precise mechanism by which YTHDFs interferes with IBs dynamics requires further investigation. However, and considering that our data showed that METTL3 knockdown resulted in a reduction in the number of IBs with the concomitant increase in their size, it is tempting to speculate that viral genomic RNA is a structural component of IBs required for the coalescence of thesex structures. Thus, the m^6^A-mediated reduction in the intracellular levels of the HRSV gRNA exerted by YTHDF proteins results in the inhibition of IBs coalescence.

Although the role of m^6^A on HRSV replication in HeLa cells was recently described (Xue et al., 2019), our data propose a different scenario occurring in HEK293T cells. However, many of these differences could be related to the viral strain, the cellular model used and/or the multiplicity of infection used. Indeed, cell type-dependent effects of m^6^A have been reported for Kaposi’s sarcoma virus (KSHV) (Tan et al., 2017; Hesser et al., 2018; Tan and Gao, 2018). Moreover, it has been suggested that the presence of m^6^A in the incoming viral RNA could act as a molecular marker avoiding sensing by RIG-I and the activation of immune response mechanisms dependent on IFN-I (Kim et al., 2020; Lu et al., 2020). Although RIG-I expression has been reported in HEK293T cells (Mukherjee et al., 2009), this cell line has been shown to lack expression of any TLRs (Hornung et al., 2002), and therefore, the IFN-I response could be attenuated in this cellular model.

Further studies aimed to understand the relationship between m^6^A and IBs dynamics will gain insights into an important molecular mechanism essential for the viral replication of this highly prevalent virus affecting infants and the elderly.

## Data Availability Statement

The raw data supporting the conclusions of this article will be made available by the authors, without undue reservation.

## Author Contributions

MA and RS-R designed the study. LT donated the strains of HRSV. FF, AV-G, and JC performed the experiments of protein and RNA quantification. LA-P performed knockdown experiments. FV-E and AG-A designed microscopy experiments. AG-A, SR-B, JC, and MA performed microscopy experiments. FF, AV-G, and AG-A performed statistical analysis. MA, RS-R, FV-E, and LT discussed and analyzed the results. MA wrote the manuscript. MA, RS-R, and FV-E performed funding acquisition. All authors contributed to the article and approved the submitted version.

## Conflict of Interest

The authors declare that the research was conducted in the absence of any commercial or financial relationships that could be construed as a potential conflict of interest.

## Publisher’s Note

All claims expressed in this article are solely those of the authors and do not necessarily represent those of their affiliated organizations, or those of the publisher, the editors and the reviewers. Any product that may be evaluated in this article, or claim that may be made by its manufacturer, is not guaranteed or endorsed by the publisher.
